# Molecular Mechanisms, Genotype–Phenotype Correlations and Patient-Specific Treatments in Inherited Metabolic Diseases

**DOI:** 10.3390/jpm13010117

**Published:** 2023-01-05

**Authors:** Angel L. Pey

**Affiliations:** Departamento de Química Física, Unidad de Excelencia de Química Aplicada a Biomedicina y Medioambiente e Instituto de Biotecnología, Facultad de Ciencias, Universidad de Granada, Av. Fuentenueva s/n, 18071 Granada, Spain; angelpey@ugr.es; Tel.: +34-958243173; Fax: +34-958272879

Advances in DNA sequencing technologies are revealing a vast genetic heterogeneity in human population, which may predispose to metabolic alterations if the activity of metabolic enzymes is affected [1,2,3,4]. Mutations and polymorphism may affect several protein functions simultaneously, such as catalysis, regulation, ligand binding, intracellular folding, degradation, aggregation and transport [5,6,7,8,9,10,11,12]. This is a consequence of the propagation of local stability effects across the protein structure [11,13,14,15,16,17,18] (Figure 1). In this Special Issue, we focus on three critical aspects currently under development regarding metabolic diseases with a genetic origin, namely the high-throughput computation of genotype–phenotype correlations [19], their treatment using natural or pharmacological chaperones [20,21] and the inhibition of altered metabolic routes to prevent the accumulation of toxic intermediates [22].

Most human proteins are oligomeric. Human alanine:glyoxylate aminotransferase (AGT) is responsible for glyoxylate detoxification in human liver peroxisomes, and inherited mutations lead to a life-threatening metabolic disease called primary hyperoxaluria type I (PH1), characterized by oxalate accumulation and liver and kidney failure [11,23]. Dindo and coworkers describe in this Special Issue the importance of the dimerization of human alanine:glyoxylate amino transferase (AGT) for the proper folding of the enzyme in cells and its import to peroxisomes, where the enzyme is metabolically useful, as well as the chaperone role of the protein cofactor pyridoxal 5′-phosphate (PLP) for dimerization and function [24]. More recently, the same group have described the successful development of pharmacological chaperones that partially restore the normal AGT activity of PH1-causing mutations [25]. Moya-Garzón and coworkers present in this Special Issue an alternative for the treatment of PH1 based on inhibitors of oxalate formation that is currently under further development by using tools from medicinal chemistry [22,26]. Human galactose 1-phosphate uridylyltransferase (GALT) is also a dimeric protein involved in the metabolism of galactose, and whose deficiency due to inherited mutations leads to galactosemia type I (GT1) [27]. The recently reported structure for GALT has allowed us to rationalize the effect of many disease-causing mutations, although many aspects of GT1 pathophysiology remain unclear [27]. The pathological mechanisms as well as novel therapies (mechanism- or phenomenological-based) and disease models are extensively discussed for GT1 in this Special Issue by Banford and coworkers [28] and Delnoy and coworkers [29].

Another example of oligomeric protein with a very complex regulation (through product inhibition and several phosphorylation events at the N-terminal domain), for which structural information has been recently provided is Tyrosine hydroxylase (TH) [30], the rate-limiting enzyme in catecholamine biosynthesis. The recently available high-resolution structural information for TH will likely improve our capacity to predict functional or folding effects of inherited mutations in the TH associated with Dopa-responsive dystonia (DRD), DA deficiency and Parkinsonisms [30]. In this Special Issue, Nygaard and coworkers deeply discussed multiple aspects of DRD, including genotype–phenotype correlations based on structural and experimental evidence as well as different therapeutic approaches such as pharmacological chaperones, gene- and enzyme-replacement therapies [31].

Pharmacophores found in high-throughput screening campaigns are promising as pharmacological chaperones for the treatment of inherited metabolic disease, although they often show drawbacks regarding solubility, bioavailability and side-effects [21,32,33]. In this Special Issue, Bernardo-Seisdedos and coworkers described the improvement of Ciclopirox as a repurposed drug for the treatment Congenital Erythropoietic Porphyria (CEP), a disease caused by a deficiency in the UROIIIS protein [7,33,34]. This type of optimization is fundamental to bringing basic studies to the clinical realm.

Overall, this Special Issue addresses several fundamental questions on the prediction of phenotypes and novel therapies for Personalized Medicine in Inborn Errors of Metabolism. It is important to highlight that all the groups that have contributed to this Special Issue are currently working on the improvement of the different mechanistic and therapeutic approaches presented. The exception is Prof. David J. Timson, who sadly passed away last summer. We hope that we will update all these studies in 2023.

## Figures and Tables

**Figure 1:**
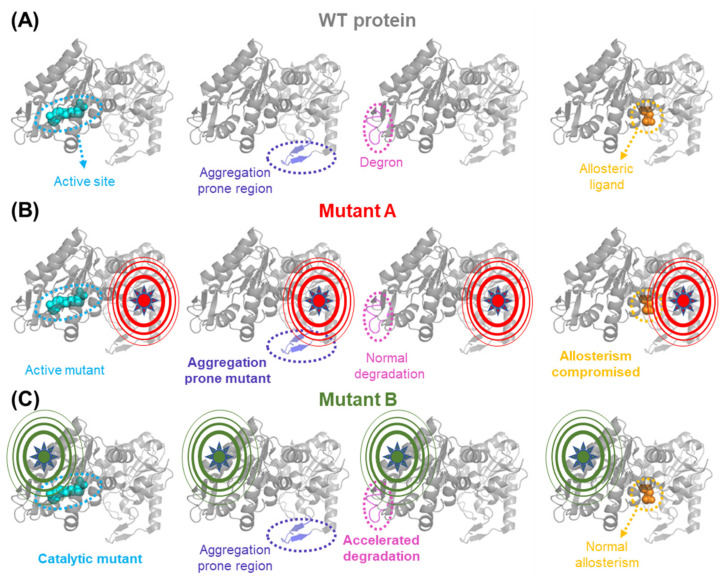
Long-range propagation of mutational effects causes pleiotropic effects on protein functional features. (**A**) The WT form of a protein contains different functional sites: an active site, a region whose conformation determines protein aggregation, a flexible degron that controls protein degradation and a regulatory binding site for an allosteric ligand. (**B**,**C**) Pleiotropic effects of two model mutations destabilizing different regions of the protein. Note that the propagation of mutational effects in mutant A enhances protein aggregation and affects the regulatory binding site, while mutant B affects active site performance and accelerates protein degradation.

## References

[1] McInnes G., Sharo A.G., Koleske M.L., Brown J.E.H., Norstad M., Adhikari A.N., Wang S., Brenner S.E., Halpern J., Koenig B.A. (2021). Opportunities and Challenges for the Computational Interpretation of Rare Variation in Clinically Important Genes. Am. J. Hum. Genet..

[2] Lek M., Karczewski K.J., Minikel E.V., Samocha K.E., Banks E., Fennell T., O’Donnell-Luria A.H., Ware J.S., Hill A.J., Cummings B.B. (2016). Analysis of Protein-Coding Genetic Variation in 60,706 Humans. Nature.

[3] Manolio T.A., Fowler D.M., Starita L.M., Haendel M.A., MacArthur D.G., Biesecker L.G., Worthey E., Chisholm R.L., Green E.D., Jacob H.J. (2017). Bedside Back to Bench: Building Bridges between Basic and Clinical Genomic Research. Cell.

[4] Pacheco-Garcia J.L., Cagiada M., Tienne-Matos K., Salido E., Lindorff-Larsen K., Pey L.A. (2022). Effect of Naturally-Occurring Mutations on the Stability and Function of Cancer-Associated NQO1: Comparison of Experiments and Computation. Front. Mol. Biosci..

[5] Flydal M.I., Martinez A. (2013). Phenylalanine Hydroxylase: Function, Structure, and Regulation. IUBMB Life.

[6] Hnízda A., Majtan T., Liu L., Pey A.L., Carpenter J.F., Kodíček M., Kožich V., Kraus J.P. (2012). Conformational Properties of Nine Purified Cystathionine β-Synthase Mutants. Biochemistry.

[7] Blouin J.-M., Bernardo-Seisdedos G., Sasso E., Esteve J., Ged C., Lalanne M., Sanz-Parra A., Urquiza P., de Verneuil H., Millet O. (2017). Missense UROS Mutations Causing Congenital Erythropoietic Porphyria Reduce UROS Homeostasis That Can Be Rescued by Proteasome Inhibition. Hum. Mol. Genet..

[8] Rivera-Barahona A., Navarrete R., García-Rodríguez R., Richard E., Ugarte M., Pérez-Cerda C., Pérez B., Gámez A., Desviat L.R. (2018). Identification of 34 Novel Mutations in Propionic Acidemia: Functional Characterization of Missense Variants and Phenotype Associations. Mol. Genet. Metab..

[9] Salido E., Pey A.L., Rodriguez R., Lorenzo V. (2012). Primary Hyperoxalurias: Disorders of Glyoxylate Detoxification. Biochim. Biophys. Acta Mol. Basis Dis..

[10] Pey A.L., Padín-Gonzalez E., Mesa-Torres N., Timson D.J. (2014). The Metastability of Human UDP-Galactose 4′-Epimerase (GALE) Is Increased by Variants Associated with Type III Galactosemia but Decreased by Substrate and Cofactor Binding. Arch. Biochem. Biophys..

[11] Medina-Carmona E., Betancor-Fernández I., Santos J., Mesa-Torres N., Grottelli S., Batlle C., Naganathan A.N., Oppici E., Cellini B., Ventura S. (2019). Insight into the Specificity and Severity of Pathogenic Mechanisms Associated with Missense Mutations through Experimental and Structural Perturbation Analyses. Hum. Mol. Genet..

[12] Pacheco-Garcia J.L., Anoz-Carbonell E., Vankova P., Kannan A., Palomino-Morales R., Mesa-Torres N., Salido E., Man P., Medina M., Naganathan A.N. (2021). Structural Basis of the Pleiotropic and Specific Phenotypic Consequences of Missense Mutations in the Multifunctional NAD(P)H:Quinone Oxidoreductase 1 and Their Pharmacological Rescue. Redox Biol..

[13] Naganathan A.N. (2019). Modulation of Allosteric Coupling by Mutations: From Protein Dynamics and Packing to Altered Native Ensembles and Function. Curr. Opin. Struct. Biol..

[14] Rajasekaran N., Naganathan A.N. (2017). A Self-Consistent Structural Perturbation Approach for Determining the Magnitude and Extent of Allosteric Coupling in Proteins. Biochem. J..

[15] Rajasekaran N., Sekhar A., Naganathan A.N. (2017). A Universal Pattern in the Percolation and Dissipation of Protein Structural Perturbations. J. Phys. Chem. Lett..

[16] Rajasekaran N., Suresh S., Gopi S., Raman K., Naganathan A.N. (2017). A General Mechanism for the Propagation of Mutational Effects in Proteins. Biochemistry.

[17] Pacheco-García J.L., Loginov D.S., Naganathan A.N., Vankova P., Cano-Muñoz M., Man P., Pey A.L. (2022). Loss of Stability and Unfolding Cooperativity in HPGK1 upon Gradual Structural Perturbation of Its N-Terminal Domain Hydrophobic Core. Sci. Rep..

[18] Pacheco-Garcia J.L., Loginov D.S., Anoz-Carbonell E., Vankova P., Palomino-Morales R., Salido E., Man P., Medina M., Naganathan A.N., Pey A.L. (2022). Allosteric Communication in the Multifunctional and Redox NQO1 Protein Studied by Cavity-Making Mutations. Antioxidants.

[19] Stein A., Fowler D.M., Hartmann-Petersen R., Lindorff-Larsen K. (2019). Biophysical and Mechanistic Models for Disease-Causing Protein Variants. Trends Biochem. Sci..

[20] Martinez A., Calvo A.C., Teigen K., Pey A.L. (2008). Chapter 3 Rescuing Proteins of Low Kinetic Stability by Chaperones and Natural Ligands. Phenylketonuria, a Case Study.

[21] Pey A.L., Ying M., Cremades N., Velazquez-Campoy A., Scherer T., Th??ny B., Sancho J., Martinez A. (2008). Identification of Pharmacological Chaperones as Potential Therapeutic Agents to Treat Phenylketonuria. J. Clin. Investig..

[22] Moya-Garzon M.D., Rodriguez-Rodriguez B., Martin-Higueras C., Franco-Montalban F., Fernandes M.X., Gomez-Vidal J.A., Pey A.L., Salido E., Diaz-Gavilan M. (2022). New Salicylic Acid Derivatives, Double Inhibitors of Glycolate Oxidase and Lactate Dehydrogenase, as Effective Agents Decreasing Oxalate Production. Eur. J. Med. Chem..

[23] Fernández-Higuero J.Á., Betancor-Fernández I., Mesa-Torres N., Muga A., Salido E., Pey A.L. (2019). Structural and Functional Insights on the Roles of Molecular Chaperones in the Mistargeting and Aggregation Phenotypes Associated with Primary Hyperoxaluria Type I. Advances in Protein Chemistry and Structural Biology.

[24] Dindo M., Ambrosini G., Oppici E., Pey A.L., O’Toole P.J., Marrison J.L., Morrison I.E.G., Butturini E., Grottelli S., Costantini C. (2021). Dimerization Drives Proper Folding of Human Alanine:Glyoxylate Aminotransferase But Is Dispensable for Peroxisomal Targeting. J. Pers. Med..

[25] Grottelli S., Annunziato G., Pampalone G., Pieroni M., Dindo M., Ferlenghi F., Costantino G., Cellini B. (2022). Identification of Human Alanine-Glyoxylate Aminotransferase Ligands as Pharmacological Chaperones for Variants Associated with Primary Hyperoxaluria Type 1. J. Med. Chem..

[26] Moya-Garzon M.D., Gomez-Vidal J.A., Alejo-Armijo A., Altarejos J., Rodriguez-Madoz J.R., Fernandes M.X., Salido E., Salido S., Diaz-Gavilan M. (2021). Small Molecule-Based Enzyme Inhibitors in the Treatment of Primary Hyperoxalurias. J. Pers. Med..

[27] McCorvie T.J., Kopec J., Pey A.L., Fitzpatrick F., Patel D., Chalk R., Shrestha L., Yue W.W. (2016). Molecular Basis of Classic Galactosemia from the Structure of Human Galactose 1-Phosphate Uridylyltransferase. Hum. Mol. Genet..

[28] Banford S., McCorvie T.J., Pey A.L., Timson D.J. (2021). Galactosemia: Towards Pharmacological Chaperones. J. Pers. Med..

[29] Delnoy B., Coelho A.I., Rubio-Gozalbo M.E. (2021). Current and Future Treatments for Classic Galactosemia. J. Pers. Med..

[30] Bueno-Carrasco M.T., Cuéllar J., Flydal M.I., Santiago C., Kråkenes T.-A., Kleppe R., López-Blanco J.R., Marcilla M., Teigen K., Alvira S. (2022). Structural Mechanism for Tyrosine Hydroxylase Inhibition by Dopamine and Reactivation by Ser40 Phosphorylation. Nat. Commun..

[31] Nygaard G., Szigetvari P.D., Grindheim A.K., Ruoff P., Martinez A., Haavik J., Kleppe R., Flydal M.I. (2021). Personalized Medicine to Improve Treatment of Dopa-Responsive Dystonia-A Focus on Tyrosine Hydroxylase Deficiency. J. Pers. Med..

[32] Segovia-Falquina C., Vilas A., Leal F., del Caño-Ochoa F., Kirk E.P., Ugarte M., Ramón-Maiques S., Gámez A., Pérez B. (2022). A Functional Platform for the Selection of Pathogenic Variants of PMM2 Amenable to Rescue via the Use of Pharmacological Chaperones. Hum. Mutat..

[33] Urquiza P., Laín A., Sanz-Parra A., Moreno J., Bernardo-Seisdedos G., Dubus P., González E., Gutiérrez-de-Juan V., García S., Eraña H. (2018). Repurposing Ciclopirox as a Pharmacological Chaperone in a Model of Congenital Erythropoietic Porphyria. Sci. Transl. Med..

[34] Bernardo-Seisdedos G., Charco J.M., SanJuan I., García-Martínez S., Urquiza P., Eraña H., Castilla J., Millet O. (2021). Improving the Pharmacological Properties of Ciclopirox for Its Use in Congenital Erythropoietic Porphyria. J. Pers. Med..

